# Single-cell genomics and spatial transcriptomics: Discovery of novel cell states and cellular interactions in liver physiology and disease biology

**DOI:** 10.1016/j.jhep.2020.06.004

**Published:** 2020-06-10

**Authors:** Antonio Saviano, Neil C. Henderson, Thomas F. Baumert

**Affiliations:** 1 Inserm, U1110, Institut de Recherche sur les Maladies Virales et Hépatiques, Strasbourg, France; 2 Université de Strasbourg, Strasbourg, France; 3 Pôle Hépato-digestif, Institut-Hospitalo-Universitaire, Hôpitaux Universitaires de Strasbourg, Strasbourg, France; 4 Centre for Inflammation Research, University of Edinburgh, Edinburgh EH16 4TJ, UK; 5 MRC Human Genetics Unit, Institute of Genetics and Molecular Medicine, University of Edinburgh, Edinburgh EH4 2XU, UK; 6 Institut Universitaire de France, Paris, France

**Keywords:** Single-cell, Single-cell RNA sequencing, Spatial transcriptomics, Zonation, Liver diseases, Hepatocellular carcinoma, Microenvironment, Cirrhosis, Fibrosis, Non-parenchymal cells

## Abstract

Transcriptome analysis enables the study of gene expression in human tissues and is a valuable tool to characterise liver function and gene expression dynamics during liver disease, as well as to identify prognostic markers or signatures, and to facilitate discovery of new therapeutic targets. In contrast to whole tissue RNA sequencing analysis, single-cell RNA-sequencing (scRNA-seq) and spatial transcriptomics enables the study of transcriptional activity at the single cell or spatial level. ScRNA-seq has paved the way for the discovery of previously unknown cell types and subtypes in normal and diseased liver, facilitating the study of rare cells (such as liver progenitor cells) and the functional roles of non-parenchymal cells in chronic liver disease and cancer. By adding spatial information to scRNA-seq data, spatial transcriptomics has transformed our understanding of tissue functional organisation and cell-to-cell interactions *in situ.* These approaches have recently been applied to investigate liver regeneration, organisation and function of hepatocytes and non-parenchymal cells, and to profile the single cell landscape of chronic liver diseases and cancer. Herein, we review the principles and technologies behind scRNA-seq and spatial transcriptomic approaches, highlighting the recent discoveries and novel insights these methodologies have yielded in both liver physiology and disease biology.

## Introduction

Sequencing technologies are increasingly used to study phenotypes and drivers of liver disease. Whole tissue RNA sequencing has primarily been used to identify major differences in gene expression between normal and diseased conditions. Advanced computational analyses have established gene signatures to predict patients’ prognosis and classify primary liver cancers,^1,2^ but these tools have yet to be fully integrated into clinical practice. Whole tissue RNA sequencing provides an average readout of the RNA content of a sample, which represents mixed RNA signals from the different cells present within the tissue and is thus significantly influenced by cell type prevalence. This approach cannot be used to study rare cell populations, cellular heterogeneity (*i.e.* cell subsets among major cell types), specific pathogenic cell subpopulations, or to dissect cancer clonal evolution and microenvironment. In the era of immunotherapy and precision medicine, higher resolution sequencing data are required to characterise heterogeneous tissues and complex diseases such as chronic liver disease and cancer.

**Fig. 1:**
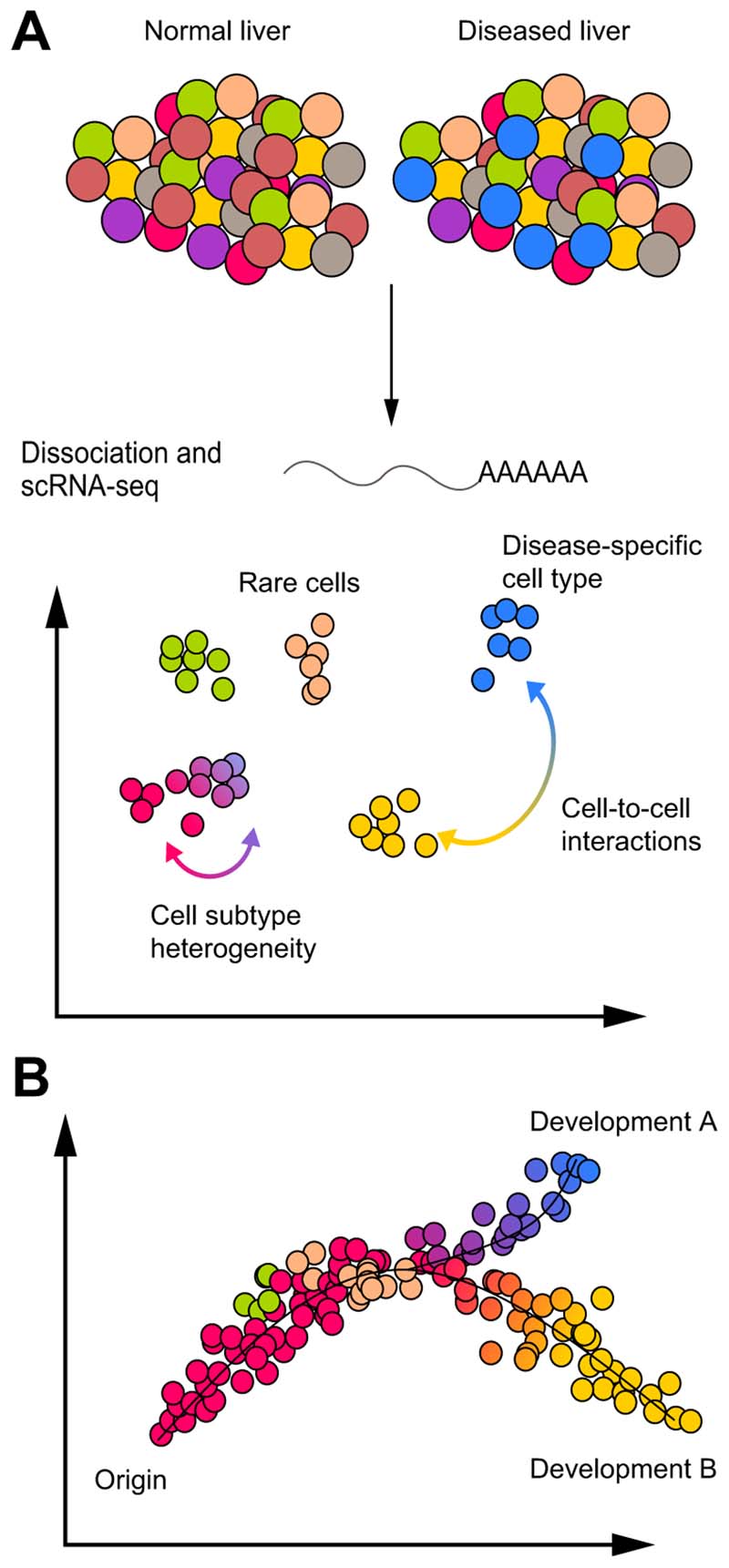
Single-cell RNA-sequencing analyses to study liver pathophysiology. (A) Normal and/or diseased liver tissue are dissociated into a single cell suspension and scRNA-seq is performed. Thousands of transcripts per cell are compressed in a 2D space where each cell is a dot and the distance between cells is a function of their similarity. Cells are can be aggregated in clusters or groups of clusters plotted as different colours and potentially representing cell types or subtypes. ScRNA-seq allows the study of rare cell types, cell state and subtype heterogeneity, disease-specific cell type and cell-to-cell interactions via ligand-receptor analysis. (B) Computational analyses such as pseudo-time diffusion mapping or RNA velocity, which analyse cell similarity and diversity, consent to trace differentiation processes, clonal evolution and cell state transitions of a specific cell type or between different cell types (from cell of origin to development A or B). ScRNA-seq, single-cell RNA-sequencing.

**Fig. 2:**
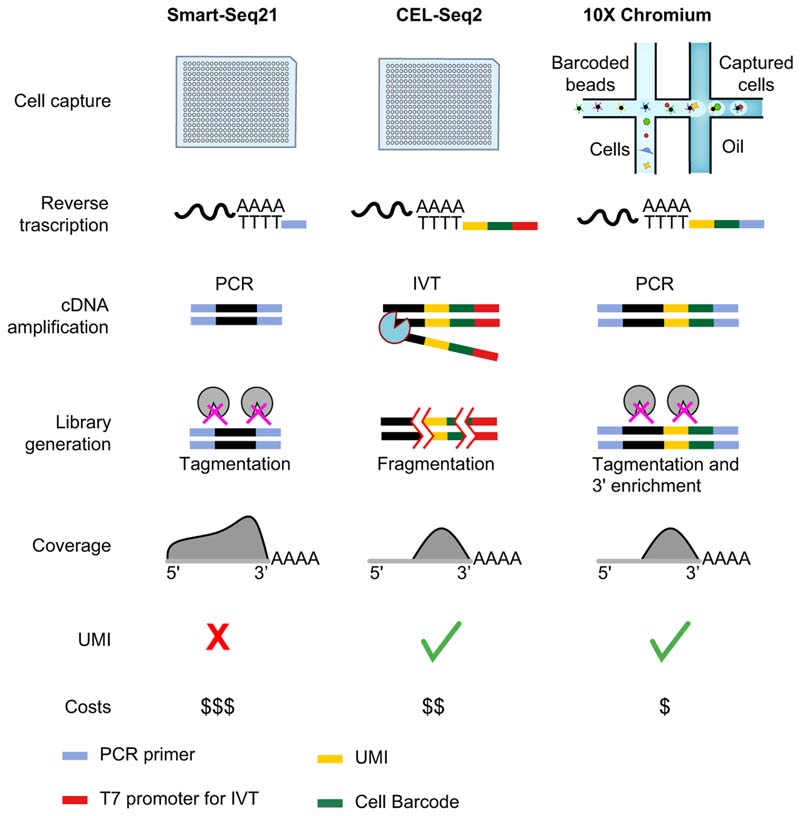
Main steps in scRNA-seq workflows and comparison of the most widely used protocols. Smart-seq2 and CEL-Seq2 are performed in 96 or 384-well plates after FACS sorting, while droplet systems (*e.g*. 10X Chromium and Drop-Seq) couple cells with barcoded beads containing UMI and primers forming water-in-oil droplets via a continuous oil flow. Reverse transcription and cDNA amplification are performed by PCR in Smart-Seq and 10X Chromium and by IVT in CEL-Seq2. In CEL-Seq2 and 10X Chromium protocols, UMI and cell-specific barcodes are added during reverse transcription to allow the pooling of the subsequent steps. Libraries are prepared by fragmentation in CEL-Seq2 and by tagmentation with or without 3'enrichment in Smart-Seq2 and 10X Chromium. Gene coverage is full-length in Smart-seq2 whereas in CEL-Seq2 and 10X Chromium only the 3′ part of the gene is sequenced. IVT, in vitro transcription; scRNA-seq, single-cell RNA-sequencing; UMI, unique molecule identifier.

Recent technological advances enabled genome-wide RNA profiling in individual cells, a technique termed single-cell RNA sequencing (scRNA-seq).^3–6^ In scRNA-seq, liver tissue is dissociated, single cells captured, and RNA sequencing is performed using several workflows Fig. 1, 2). ScRNA-seq generates very large datasets of thousands of gene transcripts per cell. These datasets are usually represented in a compressed 2D space, *e.g.* t-distributed stochastic neighbour embedding (*t*-SNE) map,^7^ where each cell is a dot and the distance between cells is a function of their similarity (Fig. 1A). In this 2D space, cells can be clustered according to their similarity and single or multiple genes can be plotted on separate *t*-SNE maps. ScRNA-seq enables the discovery, identification and/or study of rare cell types, cell subtypes, disease-specific cell types and cell-to-cell interactions via ligand-receptor expression analysis (Fig. 1A). Furthermore, computational analyses, such as pseudo-time diffusion mapping^8^ or RNA velocity,^9^ allow *in silico* lineage tracing and analysis of developmental trajectories between cell types (*e.g.* from progenitor cells to differentiated hepatocytes) or among cell subtypes (*e.g.* from cytotoxic to exhausted T cells) (Fig. 1B).

A major challenge of scRNA-seq data is to match the RNA profile of a cell with its position within a tissue (*i.e.* spatial information). This is particularly important in liver biology because the liver is spatially organised in functional lobules and acini.^10^ To address this need, spatially resolved RNA sequencing, paired-cell sequencing, complex computational algorithms and direct spatial transcriptomic techniques – in which scRNA-seq is performed on tissue sections using spatially organised RNA capture probes – have recently been developed.

Herein, we summarise and discuss the technical principles of scRNA-seq and spatial transcriptomic approaches, as well as reviewing their application and discoveries regarding liver organisation, regeneration, and cell-cell interactions in chronic liver disease and cancer.

## From liver tissue to single-cell RNA sequencing

The initial steps in a scRNA-seq experiment involve tissue dissociation and isolation of single cells which can be obtained by a variety of methods, such as FACS, magnetic separation using specific antibodies, chip-based or microdroplet-based microfluidic technologies, micromanipulation using an inverted microscope and a motorised micromanipulation platform or laser microdissection.^11^ FACS is one of the most widely used techniques and allows the selection of specific cell populations from heterogeneous tissues. High-throughput microdroplet-based microfluidic technologies (*e.g.* 10X Chromium) are increasingly used because of high capture efficiency and low costs. Microfluidic technologies are based on the dispersion of single cells into water-in-oil droplets, containing uniquely barcoded beads and primers, using a continuous oil flow as depicted in Fig. 2. The choice of single-cell capture method greatly depends on the cell types of interest, their prevalence in the tissue, and costs.

After cell isolation, scRNA-seq libraries are generated by cell lysis, reverse transcription into complementary DNA (cDNA), second-strand synthesis and cDNA amplification by PCR or *in vitro* transcription followed by deep sequencing. These steps vary across the different scRNA-seq protocols (Fig. 2). Smart-seq2 is a protocol which uses template-switching technologies for the reverse transcription and PCR technologies for the amplification, enabling the sequencing of full-length transcripts and the study of splicing events and allele-specific expression.^6,12,13^ Smart-seq2 is limited by high costs, so different protocols have evolved to allow for adequate RNA coverage and reduced costs. These protocols involve the capture of the RNA poly(A) tail with the insertion into the cDNA of random unique molecular identifiers (UMIs) and pre-specified cellular barcodes (Fig. 2). The presence of both cellular barcodes and UMIs in each single cDNA enables pooling of cDNAs from different cells for the amplification and sequencing steps, significantly reducing the costs per run. The cell of origin is inferred using the cellular barcodes and gene expression is quantified by counting and normalising UMIs per single cells. In terms of performance, Smart-seq2 and CEL-seq2 showed the highest sensitivity, while Drop-seq is less expensive but has lower capture efficiency and resolution.^3^ Among the different microdroplet-based microfluidic technologies, 10X Chromium results in higher sensitivity and less technical noise.^14^ Finally, the combination of multiple scRNA-seq techniques, *e.g.* a microdroplet-based system and Smart-Seq2, can be synergistic, increasing the probability of capturing both rare cell types and low abundance transcripts.^15^

ScRNA-seq comprises multiple technologies and the choice of platform used should be guided by the biological question. The appropriate technique or combination of techniques should be chosen in the context of the study design and endpoints required (*e.g.* study of rare cell types or lowly expressed genes or splicing variant analysis). Smart-seq2 is preferred when analysing splicing, transcriptome annotations or genome integrations while high-throughput microdroplet-based microfluidic technologies are preferred for broader cell coverage at shallower sequencing read depths.

## Liver physiology at the single-cell level

### Rewind the tape: Gather spatial information from single-cell data to study liver zonation

**Fig. 3:**
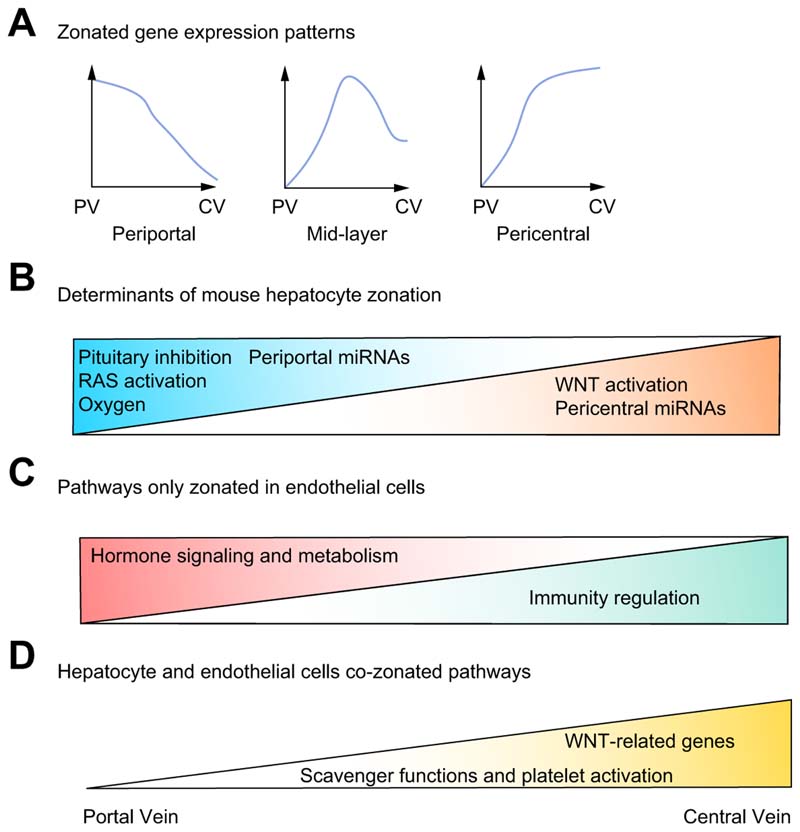
New concepts in liver zonation derived from scRNA-seq studies. (A) Zonated genes can have a non-monotonic pattern with genes peaking in the mid-layers of liver lobule. B) Determinants of mouse hepatocyte zonation on periportal (left) and pericentral genes (right). C-D) LSEC specific zonated pathways. Periportal LSECs are enriched in pathways related to hormone signalling and metabolism while pericentral LSECs are enriched in immune regulatory genes, WNT-related genes, platelet activation and scavenger function pathways. LSECs and hepatocytes show co-zonation in pericentral areas of WNT-related genes (mouse data) and platelet activation and scavenger function pathways (human data). LSEC(s), liver sinusoidal endothelial cells; MiRNA, microRNA; scRNA-seq, single-cell RNA-sequencing.

One of the first applications of scRNA-seq has been the study of liver zonation in mice and humans. The liver is a highly organised tissue, and the porto-central axis of the acinus is a fundamental functional unit during homeostasis and disease development. Hepatocyte function varies along this axis, with hepatocytes classically divided into 3 zones. A major challenge in the use of scRNA-seq for the study of liver physiology is the integration of individual cell RNA data with spatial information. To overcome this hurdle, specific sequencing strategies and bioinformatic analyses have been developed (Table 1), allowing new insights into liver zonation (Fig. 3). Halpern *et al.* studied liver zonation in mice by combining scRNA-seq with single-molecule RNA fluorescence *in situ* hybridisation (smRNA-FISH) to perform spatially resolved RNA-sequencing.^16^ At first, they used smRNA-FISH to assess, at high-resolution, the spatial distribution of known zonated landmark genes, allowing their fine porto-central profiling. Then scRNA-seq of mouse hepatocytes was performed and the porto-central profile of landmark genes was used to assign a porto-central position to each single cell (for review see^17^). Spatially resolved scRNA-seq data from the mouse liver demonstrated that i) major determinants of liver zonation were not only oxygen gradient and WNT signalling,^18^ but also RAS signalling, which activates periportal genes, and pituitary signals, which inhibit periportal genes (Fig. 3B); ii) zonation is not always monotonic and some genes, *e.g. Hamp* encoding for hepcidin, have the highest expression in the mid-layers of the lobule (Fig. 3A); iii) genes encoding for enzymes involved in bile acid metabolism are differently expressed along the porto-central axis, suggesting the spatial zonation of entire metabolic processes; iv) metabolites produced in periportal areas are taken up by pericentral hepatocytes in a process called spatial recycling.

Once the spatial transcript data of a certain cell type is known, paired-cell sequencing is an elegant technique to infer the zonation of other cell types and to identify strong cell-to-cell interactions (for review see^17^). Halpern and colleagues sequenced doublets of hepatocytes and liver sinusoidal endothelial cells (LSECs) and used hepatocyte single-cell zonation data^16^ to infer the zonation of LSECs.^19^ This analysis showed that LSEC genes are significantly zonated, and pericentral LSECs are enriched with WNT signalling genes and modulators, which are major determinants of hepatocyte zonation, suggesting that LSECs might shape hepatocyte zonation.

When surface proteins are available as spatial markers, spatial sorting with FACS can be used to sort cells from a specific area. Combining ≥2 inversely zonated markers enables the sorting of cells from specific liver lobule areas, facilitating not only scRNA-seq but also multi-omics analyses.^20^ Mass spectrometry proteomics and RNA-seq on spatially sorted hepatocytes enabled mapping of protein zonation and the correlation of gene expression with protein expression in specific liver zones. Bulk microRNA (miRNA) microarray measurement after spatial sorting on mouse livers revealed that miRNAs are zonated along the porto-central axis.^20^ MiRNAs are short non-coding RNA oligonucleotides which target specific messenger RNAs to increase their degradation or to decrease their translation.^26^ Forty-five percent of known and validated hepatocyte miRNAs were found to be mildly pericentral zonated (79%) or strongly periportal zonated (11%)^20^ with their targets inversely zonated. The study of mouse miRNA zonation via spatial sorting revealed their inverse correlation with WNT-related genes, suggesting a potential role of miRNAs in determining hepatocyte zonation.

Computational analysis can also help with inference of spatial information from scRNA-seq data when spatial organisation is the main source of heterogeneity in a tissue (Table 1). Aizarani *et al.* applied diffusion pseudotime analysis to model zonation of hepatocytes and LSECs in healthy human livers.^21^ This computational analysis was able to i) profile for the first time, at a single-gene level, the porto-central zonation of human hepatocytes and LSECs; ii) demonstrate that LSEC genes are highly zonated and iii) demonstrate that both hepatocytes and LSECs have genes with non-monotonic zonation patterns. More than 60% of LSEC genes were found to be zonated: periportal LSECs were enriched in genes involved in hormone signalling and metabolism (*e.g.* incretin and angiotensinogen metabolism) while central/mid LSECs were enriched in genes involved in platelet activation, immunity regulation and scavenger functions. Interestingly, scavenger and platelet activation genes were also enriched in central/mid-zone hepatocytes, suggesting a functional co-zonation of hepatocytes and LSECs (Fig. 3C,D).

More complex computational algorithms, enabling spatial information to be inferred from scRNA-seq data, have recently been developed. NovoSpaRc is a computational framework allowing *de novo* spatial reconstruction of single-cell gene expression cartographies with or without the use of known spatial information and marker genes.^22^ NovoSpaRc assumes that physically apposed cells probably share similar transcriptomic profiles and that physical distance can be a function of transcriptomic difference. The algorithm can reconstruct, in a virtual space, the organisation of symmetric tissues, *e.g.* normal liver and intestine, but also early embryos and charts of complex tissues such as the cerebellum and kidney. However, novoSpaRc has not yet been used to investigate liver spatial organisation and function.

**Fig. 4:**
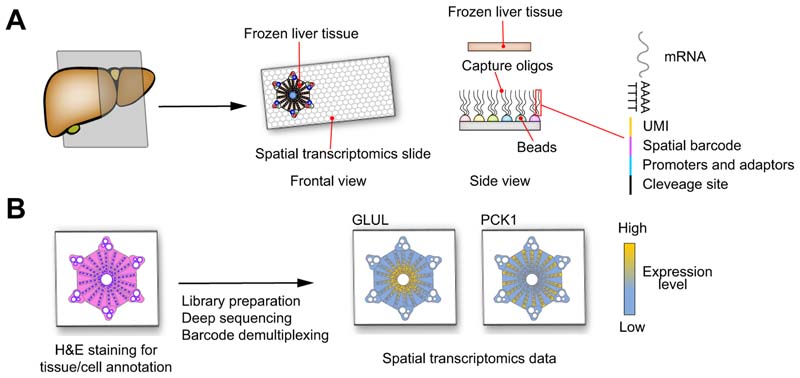
Model of in *situ* spatial transcriptomics. (A) Frozen liver tissue is cut and placed on a special slide special slide covered by beads carrying capture oligos composed by a polyd(T) tail for RNAs capture, a spatial barcode defining bead position, an UMI for transcript count, promoters and adaptors for cDNA synthesis, amplification and sequencing and a cleavage site to detach the oligos from the slide. B) The liver tissue on the spatial transcriptomics slide is fixed, stained by H&E and scanned by a conventional microscopy slide scanner. The tissue is lysed to release RNA, the capture oligos are cleaved and the libraries prepared as for scRNA-seq. The H&E image combined with data and coordinates of the spatial barcodes produce high-resolution single-cell gene expression data. GLUL encoding for glutamate-ammonia ligase is a known pericentral zonated gene and PCK1 encoding for phosphoenolpyruvate carboxykinase 1 is a periportal zonated gene. cDNA, complementary DNA; scRNA-seq, single-cell RNA-sequencing; UMI, unique molecule identifier.

One of the challenges of inferring spatial information from standard scRNA-seq data is the requirement for careful, follow-on validation by direct spatial techniques such as immunohistochemistry, immunofluorescence or FISH. To overcome this issue, systems which allow *in situ* spatial transcriptomics have recently been developed.^23–25^ These systems generally consist of a special slide covered by beads carrying oligos composed of a polyd(T) tail for RNA capture, a spatial barcode defining bead position, a UMI for transcript count, promoters and adaptors for cDNA synthesis, amplification and sequencing, and a cleavage site to detach the oligos from the slide (Fig. 4A). Frozen liver tissue is cut, placed on the spatial transcriptomic slide, stained by H&E and scanned by a conventional microscopy slide scanner. The tissue is lysed – releasing RNA which is captured by the oligos – the captured oligos are cleaved, and the library is prepared as for scRNA-seq. Once the sequencing is performed, the H&E image is combined with the coordinates of the spatial barcode beads to produce single-cell spatial transcriptomic data. Indeed, H&E staining provides data on cell position and size and allows for the definition of cell boundaries and the assignment of, in certain protocols, spatial barcodes to a single cell. Single-cell transcriptomic data can then be visualised in 2D space (Fig. 4B). These techniques have been successfully used to investigate complex tissues, such as the brain^23–25^ or breast cancer,^25^ and hold exceptional promise for the detailed study of liver disease.

### Using scRNA-seq to identify progenitor cells in the context of liver development and regeneration

Regeneration is one of the key features of liver physiology, but the precise identity and degree of heterogeneity of hepatobiliary precursor cells has yet to be fully clarified. Data has mainly been generated from mouse models, with differing progenitor populations (ranging from biliary-like progenitor cells to differentiated hepatocytes) proposed as the major sources of the hepatic epithelial regenerative response, depending on injury model and experimental context.^27–31^ ScRNA-seq, with its ability to help study rare cell types, has recently been used in this area to investigate heterogeneity and signalling pathways within hepatobiliary precursors in both foetal and adult livers.

Single-cell analysis of the human foetal liver has identified a distinct hepatobiliary hybrid progenitor (HHyP) cell capable of lineage commitment towards hepatocytes or biliary epithelial cells.^32^ The foetal HHyP belongs to the EPCAM^+^/NCAM^+^/TROP2^-^ compartment and showed both cholangiocyte, hepatocyte and classical progenitor markers. This cell can be found in the liver ductal plate which is a single or double layered structure of small cuboidal cells at the interface between hepatoblasts and the portal mesenchyme.

**Fig. 5:**
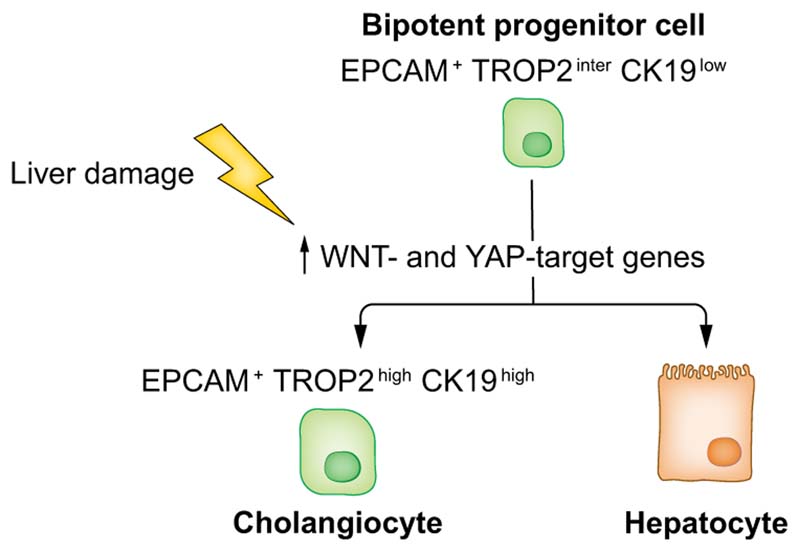
Bipotent progenitor cell in the normal human liver revealed by scRNA-seq. ScRNA-seq of human liver identified an EPCAM+TROP2^inter^ CK19^low^ progenitor cell which has the potential to differentiate into cholangiocytes or hepatocytes (human data). Upon liver damage, progenitor cells upregulate WNT-and YAP-target genes promoting liver regeneration (mouse data). ScRNA-seq, single-cell RNA-sequencing.

Aizarani and colleagues used scRNA-seq to analyse the heterogeneity across EPCAM^+^ cells in healthy human livers to understand whether the adult liver has a cell type analogous to the HHyP. They observed considerable heterogeneity within the EPCAM^+^ compartment, which comprises an EPCAM^+^TROP2^int^CK19^low^ progenitor cell with the potential to form bipotent organoids and to commit to either a hepatocyte or cholangiocyte fate^21,33^ (Fig. 5). This previously unknown adult liver progenitor cell is located in the canals of Hering and represents the equivalent of the foetal HHyP and the oval cell described in mice.^31^

In the normal liver, progenitor cells are usually quiescent and the mechanisms underlying their commitment and activation following liver injury are still unclear. In animal models, liver injury can be induced in a reproducible fashion with several strategies mimicking different liver pathologies. ScRNA-seq has been successfully applied to mouse models of liver injury to study drivers of liver regeneration, revealing YAP target genes as a major source of the heterogeneity in the EPCAM^+^ compartment.^34^ This YAP target gene signature represents a dynamic inducible state that is upregulated during liver injury, promoting and sustaining progenitor proliferation and liver regeneration (Fig. 5).^34^

In summary, scRNA-seq has facilitated the discovery of a bipotent progenitor cell in the EPCAM^+^ compartment in both foetal and normal adult liver, whose activation is associated with an upregulation of YAP target genes. To accurately characterise drivers of liver regeneration in human disease, further analyses focused on progenitor cell populations in human livers after chronic and acute injury are required.

## Novel insights into chronic liver disease and cancer microenvironment

### The phenotype of non-parenchymal cells in chronic liver disease and cirrhosis

Two human liver single-cell atlases provide a detailed insight into the composition of the normal liver – using 2 complementary sequencing techniques (miniaturised CEL-Seq2^35^ and 10X Chromium,^36^) – and constitute a reference point for single-cell based research in liver disease.^21,37^

The liver microenvironment, comprising hepatocytes and non-parenchymal cells (NPCs), plays a key role in the pathogenesis of all chronic liver diseases. In response to chronic hepatocyte damage, immune cells produce pro-inflammatory cytokines and chemokines and activate quiescent hepatic stellate cells (HSCs) which become myofibroblasts that are responsible for collagen and extracellular matrix accumulation^38,39^ - a hallmark of liver fibrosis.^40^ This dysregulation of liver immunity is common across different forms of chronic liver diseases and triggers cellular stress and death, apoptosis, liver fibrosis, and hepatocyte proliferation and liver regeneration.^38^ Single-cell studies have been carried out to uncover the heterogeneity and complex cell-to-cell interactions of NPCs in chronic liver diseases and cirrhosis.

### Liver endothelial cells are involved in multiple cell-to-cell interactions and prime differentiation of circulating monocytes into liver macrophages

A single-cell study of NPCs in healthy mice and mice with diet-induced non-alcoholic steatohepatitis (NASH) – amylin [AMLN] model^41^ – focused on the characterisation of the NPC secretome and cell-to-cell interactions.^42^ LSECs were found to secrete angiocrine factors and express several genes involved in cell-to-cell interactions. Ligands were expressed by cholangiocytes, HSCs and LSECs, suggesting extensive interactions with other NPCs as well as autocrine signalling. In NASH livers, LSECs upregulated the expression of genes implicated in lipid metabolism, chemokine release and antigen presentation, whilst genes involved in vascular homeostasis and vascular development were downregulated, inducing a significant disruption of sinusoid capillaries.^42^

LSECs are the port of entry of monocyte and other bone marrow-derived cells in the liver lobule. LSEC-to-monocyte interactions are crucial in determining the fate of circulating monocytes and their differentiation into liver macrophages.^43^ Livers of Kupffer cell (KC)-depleted mice are rapidly repopulated by circulating monocytes which acquire a KC-like phenotype. LSECs express DLL4 (delta like canonical Notch ligand 4) and TGFβ1 (transforming growth factor β1) that interact, respectively, with NOTCH and TGFβ/BMP receptors on monocytes, downregulating monocyte-specific genes. Single-cell analysis of mouse models demonstrated the expansion of monocyte-derived macrophages, with a unique inflammatory phenotype, in NASH livers.^44^ Whether dysregulated NASH LSECs determine the phenotype of the NASH monocyte-derived macrophages is still unknown and further studies are needed to elucidate LSEC-to-monocyte interactions in the context of NASH pathogenesis.

### Hepatic stellate cells are spatially and functionally zonated and are hubs of autocrine and paracrine signalling

HSCs had previously been thought to represent a functionally homogeneous population. Dobie *et al.* used scRNA-seq to deconvolve the hepatic mesenchyme in both healthy and fibrotic mouse liver, uncovering spatial zonation of HSCs across the hepatic lobule.^45^ HSCs partition into topographically diametric lobule regions, designated portal vein-associated HSCs (PaHSCs) and central vein-associated HSCs (CaHSCs). HSCs display functional zonation, with CaHSCs representing the dominant pathogenic collagen-producing cells in a mouse model of carbon tetrachloride (CCl_4_)-induced centrilobular fibrosis. Furthermore, lysophosphatidic acid receptor 1 (LPAR1) was identified as a therapeutic target on collagen-producing HSCs, and inhibition of LPAR1 resulted in decreased contractility in human HSCs *in vitro* and reduced liver fibrosis in a choline-deficient high-fat diet rodent model of NASH.^45^

ScRNA-seq from mouse livers has also shown that HSCs specifically secrete cytokines that act on LSECs, macrophages and cholangiocytes to regulate fibrotic pathways, cytokine expression, vasoactive hormone signalling and HSC apoptosis via secretion of nerve growth factor.^42^ HSCs express both *Il-11ra1,* a receptor belonging to the interleukin (IL)-6 family, and its ligand *Il-11,* constituting a previously unknown autocrine signal which stimulates the activation of STAT3 and ERK, as well as cytokine secretion.^42^ Analysis of HSC gene expression also revealed potential extrahepatic modulation of this cell type. HSCs express vasoactive hormone-responsive receptors mediating both contraction and relaxation, and specifically the effect of calcitonin gene-related peptide, parathyroid hormone and vasoactive intestinal peptide, which are not expressed by any liver cell type. In the classical view of liver fibrosis pathogenesis, HSCs are the final effector and the last step of the NPC activation cascade. Single-cell analysis of HSCs in NASH showed that they upregulate both IL-11 and cytokines, as well as modulating the function of LSECs and macrophages, suggesting a more complex bidirectional interaction. Altogether, single-cell profiling of HSCs has further extended the concept that this cell type acts as a central hub in the paracrine/autocrine network of liver NPCs in both normal and diseased liver.

### Macrophage phenotype and non-parenchymal cell interactions in the fibrotic niche

In homeostasis, the liver is continuously exposed to pathogens and toxins derived from the gut; it removes large numbers of microbes and microbe-associated molecules to maintain a tolerant and immunosuppressive environment.^46^ Data from the human liver single-cell atlases have shown that the normal liver contains not only immunomodulating macrophages with metabolic and scavenger functions, but also proinflammatory macrophages.^21,37^

In mouse models of NASH, scRNA-seq has demonstrated an expansion of macrophages with a proinflammatory phenotype. Macrophages in NASH express high levels of *Trem2* (triggering receptor expressed on myeloid cells 2) encoding an innate immunity scavenger receptor implicated in phagocytosis and clearance of apoptotic cells. This receptor has been described in the pathogenesis of Alzheimer’s disease as a microglia metabolism modifier,^47^ and in human and mouse adipose tissue macrophages in response to pathogenic lipid accumulation.^48^ Liver *Trem2^high^* macrophages were enriched in genes involved in antigen presentation, extracellular matrix remodelling, endocytosis and lysosomal degradation, suggesting an important role in NASH pathogenesis.^42^ *Trem2^high^* macrophages also overexpress *Cd9* which encodes for a tetraspanin protein involved in many cellular processes including cell differentiation, adhesion, and signal transduction,^49^ as well as preventing macrophage fusion into multinucleated giant cells.^50^ Furthermore, this NASH-associated macrophage expresses glycoprotein nmb (*Gpnmb*), a transmembrane glycoprotein that negatively regulates inflammation and was previously described in macrophages that infiltrate the liver during the recovery phase of CCl_4-_induced acute liver injury.^51,52^ *Trem2^high^* macrophages represent over 60% of KCs in NASH livers whilst they were almost undetectable in control mice, and their prevalence is reduced upon treatment with elafibranor or when switching from the AMLN diet to chow. Ramachandran and colleagues performed scRNA-seq of healthy and cirrhotic human livers and investigated heterogeneity in fibrosis-associated NPCs.^53^ Specific macrophage subpopulations were more prevalent in cirrhotic tissue and were annotated as scar-associated macrophages (SAMФ). SAMФ were marked by the expression of TREM2 and CD9, and were able to activate HSCs. Self-organising maps and pseudotime analysis at the single-cell level revealed that SAMФ are derived from blood monocytes. The differentiation process towards SAMФ fate involved the expression of genes related to antigen processing and presentation, phagocytosis, chemokines, angiogenesis, production of extracellular matrix and wound healing. SAMФ were also found in the early stages of NAFLD and in a CCl_4_ mouse model of liver fibrosis. Overall, these data suggest that TREM2^+^ SAMФ are monocyte-derived macrophages that represent a conserved innate response to chronic liver damage, promoting mesenchymal cell activation and fibrogenesis. Ongoing studies are investigating ways to manipulate this macrophage subpopulation for therapeutic gain; more functional data are required to fully understand the contribution of this novel macrophage subtype across different aetiologies of chronic liver disease.

**Fig. 6:**
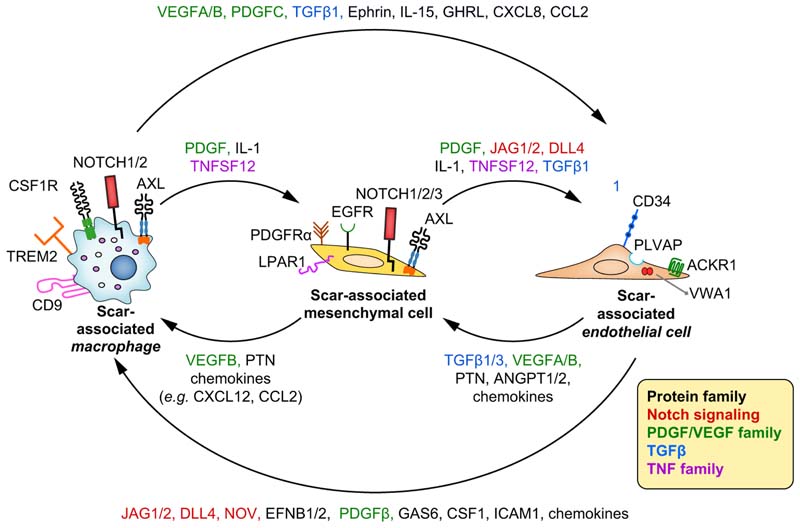
Intercellular ligand-receptor interactions in the human liver fibrotic niche. Main receptors and ligands involved in Interactions between scar-associated macrophages, scar-associated mesenchymal cells and scar-associated liver endothelial cells are presented. The most relevant molecules belong to Notch, PDGF, VEGF, TGFβ and TNF families.

ScRNA-seq analysis also unveiled the complexity of the cellular interactome of the fibrotic niche in human livers, identifying not only SAMФ but platelet-derived growth factor receptor-α (PDGFRα)^+^ mesenchymal cells (scar-associated mesenchymal cells [SAMes]) and 2 previously unknown subpopulations of scar-associated endothelial cells (SAEndo, CD34^+^PLVAP^+^VWA1^+^ and CD34^+^PLVAP^+^ACKR1^+^).^53^ Using single-cell data, multilineage ligand-receptor interaction analysis and multiplex immunofluorescence, the multidirectional interactions between SAMФ, SAEndo and SAMes were characterised (Fig. 6). Multilineage modelling of ligand-receptor interactions between these cells revealed intra-scar activity of several profibrogenic pathways including TNFRSF12A (tumour necrosis factor receptor superfamily 12A), PDGFR and NOTCH signalling. The biological relevance of the NOTCH signalling interactions in the fibrotic niche has been proven *in vitro* by coculturing primary SAEndo and HSC cells, which resulted in collagen production that is decreased upon treatment with the Notch-signalling inhibitor dibenzazepine.

In summary, scRNA-seq revealed novel scar-associated subpopulations of macrophages, endothelial cells and mesenchymal cells inhabiting the fibrotic niche in cirrhosis; this has shed light on how these different cell types interact to promote fibrosis. Fibrogenesis in cirrhosis is a highly complex process characterised by the interaction of multiple different cell lineages which are in various states of differentiation and activation. Development of novel antifibrotic therapies will require consideration of the complexity of the fibrotic niche; treatments will likely need to modulate multiple therapeutic targets simultaneously to achieve antifibrotic efficacy.

## Unravelling tumour microenvironment and heterogeneity within primary liver cancer

Aizarani *et al.* used single-cell analysis, with their normal human cell atlas as a reference, to characterise perturbed cell states in HCC.^21^ They showed that i) cancer epithelial cells upregulate proinflammatory, WNT and Hedgehog genes; ii) endothelial cells in HCC lose classical sinusoidal markers and display typical macrovascular endothelial cell markers in line with the arterialisation process, characterising HCC development; and iii) both HCC endothelial cells and macrophages downregulate pathways of innate immunity and upregulate receptor tyrosine kinase signalling, targets of the currently approved systemic treatments for HCC, such as sorafenib and regorafenib. Interestingly, HCC endothelial cells expressed *CD34* and plasmalemma vesicle associated protein (*PLVAP*) at high levels, as also observed in SAEndo,^53^ suggesting potentially common changes in fibrosis-associated and cancer-associated endothelial cells.

**Fig. 7:**
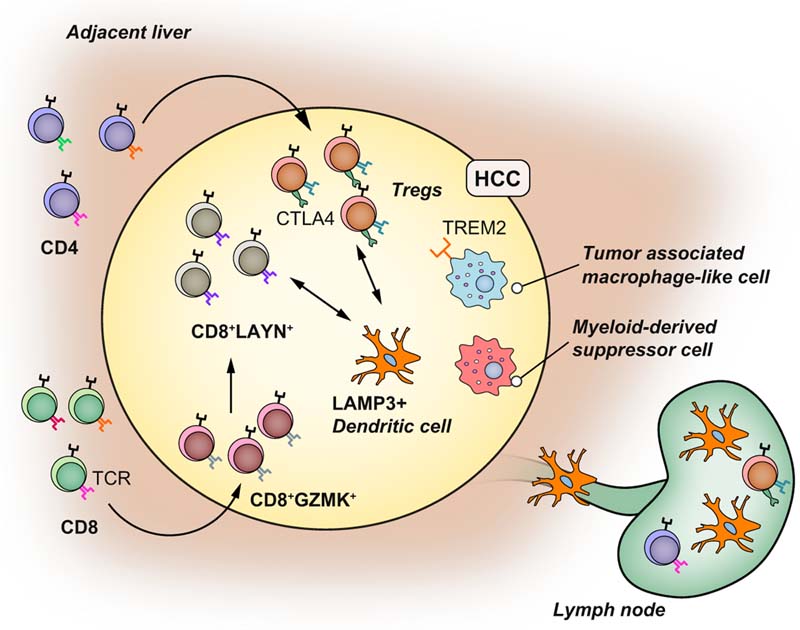
Insights into the tumour microenvironment in hepatocellular carcinoma using scRNASeq. HCC is enriched in clonal CD4^+^CTLA4^+^ Tregs and exhausted CD8^+^LAYN^+^ lymphocytes expressing the same TCR (T-cell receptor). CD8^+^LAYN^+^ lymphocytes derived from CD8^+^GMZK^+^ lymphocytes. LAMP3^+^ DCs are mature DCs enriched in HCC that interact with exhausted T cells and Tregs via the IL-15 and PD1/PD-L1 axis and can migrate into lymph nodes. The HCC microenvironment also includes myeloid-derived suppressor cells with strong immunosuppressive functions and tumour-associated macrophage-like cells which have an intermediate proinflammatory-immunosuppressive phenotype and express TREM2. DC(s), dendritic cell(s); HCC, hepatocellular carcinoma; scRNA-seq, single-cell RNA-sequencing; Tregs, regulatory T cells.

ScRNA-seq analysis has also provided new insights into the complexity of the immune cell microenvironment in HCC (Fig. 7). Zheng *et al.* investigated, at the single cell level, the T cell composition in blood, non-tumour liver and tumour tissues from patients with HCC. T regulatory cells (Tregs, CD4^+^CTLA4^+^) with immunosuppressive functions and exhausted CD8^+^ T cells were clonally enriched, with the latter predicted to originate from cytotoxic CD8^+^ T cells via an intermediate *CD8^+^GZMK^+^* T cell subtype. Layilin (LAYN), a transmembrane protein with homology to c-type lectin, was identified as a novel marker of T cell exhaustion and its expression in HCC was found to be associated with higher rates of tumour recurrence.^54^ Combining Smart-Seq2 and 10X Chromium approaches, Zhang and colleagues performed scRNA-seq of CD45^+^ immune cells from tumour, lymph nodes and ascites to characterise macrophages and dendritic cells (DCs) in HCC.^15^ Lysosomal associated membrane protein 3 (LAMP3) is a DC-specific glycoprotein induced upon DC maturation after inflammatory stimulation. Mature *LAMP3^+^* DCs were observed in both HCC and lymph nodes, and these cells were predicted to interact with T and natural killer cells via IL-15 and PD-1/PD-L1 and, importantly, they were strongly associated with T cell dysfunction.^15^ Macrophages in HCC were found to occupy 2 distinct states. Some macrophages resembled myeloid-derived suppressor cells, which have a strong immunosuppressive phenotype and can regulate the function of other immune cell types including T cells and DCs.^55,56^ A second macrophage group were similar to the tumour-associated macrophages (TAMs) described in lung cancer^57^ with a mixed proinflammatory-immunosuppressive phenotype. TAM-like macrophages express *TREM2* and *GPNMB* like the SAMФ described in fibrotic livers.

The factors shaping the tumour microenvironment (TME) in HCC are still not known. In a recent study, single-cell analysis was used to explore the interconnection between intratumor heterogeneity (ITH) and TME. Data from both HCC and intrahepatic cholangiocarcinoma showed that tumours with higher ITH have a more immunosuppressive TME, are associated with more hypoxia-related genes, higher vascular endothelial growth factor (VEGF) expression and lower long-term patient survival. Hypoxia and VEGF secretion from cancer epithelial cells seem to be the main mechanism driving ITH and TME changes in heterogenous cancers, providing a supplementary rationale for the use of anti-VEGF and anti-angiogenic drugs in the treatment of primary liver cancer.^58^

Considering the urgent need for new treatment strategies for liver cancer, scRNA-seq could help not only in the identification of new therapeutic targets but also in the development of more refined tumour classification, allowing more accurate tailoring of a patient’s treatment. Preliminary classification using scRNA-seq has already been developed,^59^ but will need prospective validation before being incorporated into routine use.

Collectively scRNA-seq has helped characterise the cellular phenotypes of various cell types within the HCC microenvironment, and has shed light on the interplay between cancer epithelial cells and TME. HCC is a complex cellular ecosystem, including clonal Tregs, clonal CD8^+^LAYN^+^ exhausted T cells, pre-exhausted CD8^+^GZMK^+^ cells, LAMP3^+^ DCs, myeloid-derived suppressor cells, TAM-like macrophages and PLVAP^+^ endothelial cells resembling the endothelial cells that inhabit the liver fibrotic niche. ScRNA-seq and spatial transcriptomic approaches will be valuable tools to help increase our understanding of the cellular and molecular mechanisms regulating the TME, which should in turn aid in the identification of novel treatment targets for hepatobiliary cancers. Furthermore, these approaches should also be informative with regard to the development of more precise tumour classification and patient stratification, thereby refining clinical trial design in this area.

## Challenges and perspectives

While scRNA-seq and the associated cutting-edge computational analyses have revolutionised the investigation of complex organs and tissues and hold great promise for enabling future discoveries in hepatology, several challenges still need to be addressed. Dissociation is a critical step that can induce transcriptomic changes^60^ and should be carefully optimised to obtain the maximum dissociation yield without inducing biases. Furthermore, scRNA-seq is expensive and the analysis of single-cell data is time consuming and requires skilled bioinformatics support. Direct spatial transcriptomic techniques can potentially overcome some of these issues but their sensitivity and validity in liver-related studies is still to be determined. Finally, technologies are rapidly moving towards the development of multi-omics single cell approaches that will allow the characterisation of proteomic, gene expression and DNA mutations in the same cell.^61^ Single-cell multi-omics will allow an even more comprehensive understanding of liver biology and disease at single-cell resolution. Efforts are needed to reduce the costs of single-cell genomics technologies, and to identify histological or radiological surrogate markers that help characterise and stratify liver disease, which in turn will help predict drug response or patient prognosis without recourse to full single-cell analysis of patient samples.

## Conclusions

ScRNA-seq is a revolutionary technique which has already been successfully applied to study the biology of healthy and diseased liver at unprecedented resolution, capturing the heterogeneity of cell types and states, as well as characterising cell-to-cell interactions. The choice of scRNA-seq approach relies on study design, endpoints and costs, and often entails a compromise between costs and gene coverage. Computational algorithms, direct spatial transcriptomics and combinations of scRNA-seq and spatial techniques enable the study of single-cell gene expression in complex, highly spatially organised tissues.

ScRNA-seq has already delivered transformative new discoveries in the understanding of liver zonation, regeneration, and the biology of chronic liver disease and cancer. Liver disease biology involves multiple cell types and complex cell-to-cell interactions, and scRNA-seq allows detailed investigation of these multicellular microenvironments. The challenge now is to fully harness and translate this new knowledge into effective novel therapeutic approaches that address the major clinical challenges in hepatology.

## Supplementary data

Supplementary data to this article can be found online at https://doi.org/10.1016/j.jhep.2020.06.004.

## Figures and Tables

**Table 1:** Spatial transcriptomics and strategies to match scRNA-seq data with spatial information.

Methods	Required input data other than scRNA-seq	Pros/Cons
Spatially-resolved RNA-seq^16^	Accurate spatial pattern of two or more marker genes	High resolution and accurate.
Paired-scRNA-seq^19^	Spatial pattern of one cell forming strong cell-to-cell interactions with the cell of interest	High resolution and accurate.
Spatial sorting analysis^20^	Known extracellular marker proteins to be used for FACS	Known extracellular marker proteins are not always available. Can be used for multi-omics analysis.
DPT analysis^21^	None	Cell diversity needs to be correlated with cell position in the tissue. Validation by histology, smRNA-FISH or other imaging techniques is needed.
Gene cartography (novoSpaRc)^22^	Optional Marker genes and general tissue organization	Cell diversity needs to be correlated with cell position in the tissue. Marker genes are optional inputs to refine the analysis. Validation by histology, smRNA-FISH or other imaging techniques is needed.
In situ spatial transcriptomics^23–25^	Slide-based system	Lower sequencing depth than classical scRNA-seq but higher spatial resolution. High costs. Not data available yet on human liver tissue.

DPT, diffusion pseudo-time; scRNA-seq, single-cell RNA-sequencing; smRNA-FISH, single-molecule RNA fluorescent in *situ* hybridization.

## Key point

ScRNA-seq and spatial transcriptomics are revolutionary techniques which allow the study of liver cell composition, physiology and disease development in unprecedented detail.

## Key point

ScRNA-seq comprises multiple technologies and the choice of platform used should be guided by the biological question, the study design and endpoints required.

## Key point

Gathering spatial information from single-cell data is challenging – several sequencing strategies and computational frameworks have been developed to overcome this issue.

## Key point

ScRNA-seq has uncovered substantial functional heterogeneity within the main liver cell lineages in health and disease, identifying zonation of multiple lineages across the liver lobule, and identification of novel progenitor populations.

## Key point

Liver zonation is not restricted to hepatocytes, but it is extended to nonparenchymal cells such as liver sinusoidal endothelial cells and stellate cells.

## Key point

ScRNA-seq of cirrhotic liver samples has enabled the study of the cellular interactome in the human liver fibrotic niche.

## Key point

Notch signalling is a central pathway involved in cell interactions in the human liver fibrotic niche.

## Key point

ScRNA-seq has uncovered cellular heterogeneity within the tumour microenvironment of primary liver cancers.

## Abbreviations

AMLN: amylin
CaHSCs: central vein-associated HSCs
CCl4: carbon tetrachloride
DLL4: delta like canonical Notch ligand 4
GPNMB: glycoprotein nmb
HCC: hepatocellular carcinoma
HHyP: hepatobiliary hybrid progenitor
HSCs: hepatic stellate cells
IL-: interleukin-
ITH: intratumor heterogeneity
KC: Kupffer cell
LAMP3: lysosomal associated membrane protein 3
LAYN: layilin
LPAR1: lysophosphatidic acid receptor 1
LSECs: liver sinusoidal endothelial cells
NASH: non-alcoholic steatohepatitis
NPCs: non-parenchymal cells
PaHSCs: portal vein-associated HSCs
PDGFRα: platelet-derived growth factor receptor-α
PLVAP: plasmalemma vesicle associated protein
SAMФ: scar-associated macrophages
SAEndo: scar-associated endothelial cells
SAMes: scar-associated mesenchymal cells
scRNA-seq: single-cell RNA sequencing
smRNA-FISH: single-molecule RNA fluorescence *in situ* hybridisation
TAMs: tumour-associated macrophages
TGFβ: transforming growth factor-β
TME: tumour microenvironment
Tregs: regulatory T cells
Trem2: triggering receptor expressed on myeloid cells 2
*t*-SNE: *t-*distributed stochastic neighbour embedding
UMI: unique molecular identifiers
VEGF: vascular endothelial growth factor
